# Round the Hospitals

**Published:** 1920-10-09

**Authors:** 


### ROUND THE HOSPITALS.

The Great Ormond Street Children's Hospital has received a charming gift, from Princess Mary, in an oil painting by Mr. Harrington Mann, which shows her in uniform. She is in her red cotton gown, with sleeves rolled up ready for work, and in cap and apron, as she appeared for the first time habited for her workaday duties in the wards. We have seldom seen a more charming portrait of the Princess, who looks thoroughly at home in her hospital garb. In order to enable Her Royal Highness to take the full training as a nurse for children a special curriculum was arranged for her by the Great Ormond Street authorities, which embraced both medical and surgical work. In her case, of course, the certificate of a fully-trained nurse was neither granted nor desired. The Princess during this "intensive training" became a great favourite with her little patients, who still talk of "Nurse Mary," and their affection was fully reciprocated, for it was quite plainly a labour of love with the Princess to carry out her duties. Sister Alexander was in charge of her training. Wherever her future lot may be cast, it is certain that Princess Mary will be a tower of strength to trained nurses all the world over, and will regard their lives from an entirely different standpoint, and with real understanding of their aims, in consequence of the happy months she spent in the wards at Great Ormond Street.

For the first time in its history the Gold Medal of the Charing Cross Hospital Medical School has been won by a woman medical student, Miss C. Cooper. This medal has been presented by the Governors each year since 1819.

At the opening of the session at the Royal Free School of Medicine for Women last week there was a crowded attendance, and after Dr. Aldrich Blake, Dean of the School, had spoken of the successes achieved during the past year, Miss Maud Royden addressed the students. Her theme was "Revolutionary Thought," in allusion to the change from the period when women were regarded somewhat in the nature of natural invalids, always suffering from megrims, hysteria and similar troubles. We understand that Miss Royden attributes the revolution which has turned women into helpful members of the community largely to the fact that women are now skilled in medicine and surgery. But surely that is an effect rather than a cause of the revolution which has taken place. If any class of women more than another is responsible for the rise in the value of woman which has taken place in the last half century, we may claim this honour for the nurses, who not only led the way in service, but have ceaselessly pointed out to their patients the ways of thinking and living which could rescue them from invalidism.

At a bazaar and *fête* opened at Parc Wern, Swansea, on September 29, in aid of the local centre of the College of Nursing, Mrs. Lloyd George gave an excellent little address calling attention to the need for more and more nurses, and her remark that more centres of the College were greatly needed in Wales evoked much applause. We understand that it is in contemplation to found a new centre during the coming winter in North Wales.

It is very much to be regretted that one of our nursing contemporaries should have felt at liberty to publish information and discussion relating to the amendment to the registration rules about which reporters at the General Nursing Council were definitely requested to keep silence. Such a breach of etiquette can only tend to discourage the Council from admitting reporters at all. Until the rules are passed and are given to the press the discussion of amendments can only be misleading.

It will be interesting to see whether there are trained nurses capable of holding the office of chief accountant in the Registrar's Department of the General Nursing Council. The tendency to engage only trained nurses, or even trained nurses "by preference" for purely lay posts may easily be carried too far. Why lure a trained nurse away from her own profession by suggesting to her to undertake accountants' duties? The only consideration which ought to weigh in appointing an accountant is knowledge of the duties she will be called on to fulfil.

We regret to announce the death of Miss Florence Goode, who was matron of the Evelina Hospital for Children for fourteen years, from 1904 until the autumn of 1918, when she retired owing to failing health. She received her children's training at the Evelina and her general training at Addenbrooke's, Cambridge. She returned to the hospital in 1902 as assistant matron, a post which she held for eighteen months before succeeding to that of matron. Her work and influence were highly appreciated at the Evelina, where she had many friends, and much regret is experienced that she lived so short a time to enjoy her well-earned time of leisure.

A great effort is to be made on October 12 and 13, by means of an Oriental bazaar at the Hove Town Hall, to put the Hove Hospital on a better financial basis. The institution has lately grown in every way and is thoroughly live in all its departments, not least that of the nursing. It needs to expand if it is to keep abreast of its opportunities for usefulness, and Hove people ought to see to it that it is not crippled for lack of means.

There is no sign at present of any revolt among British nurses at the length of their nursing course. In fact, the indications are all the other way. At

**ROUND THE HOSPITALS—(*continued*).**

King's College Hospital we learn from the report that the period of training has been increased from three to four years. All salaries of the nursing staff have been raised, and the number of nurses has undergone considerable expansion in order to reduce the working hours. Over £10,000 appears in the accounts as an increase in salaries and wages for practically the whole staff over the previous year. A certain part of this increase is due to extra wages for cleaning and repairs, but the greater part represents a permanent burden on the finances of the hospital. We look in vain in the report for any list of honours won by the nursing sisters, or any account of the progress of the training-school. Yet it is admittedly one of the most honourable missions of the voluntary hospitals to keep up by means of their costly training-schools the supply of trained nurses for the benefit of the public, and some details would surely not be amiss of the manner in which this work is carried on. This finely illustrated and well-put-together report should not lack a page or two entirely devoted to the nursing staff.

---

This is an auspicious term for the King's College for Women Department of Household and Social Science. Not only has a large group of nursing sisters entered for the year's course in preparation for the posts of Sister-Tutor and Matron, but a party of eighteen nurses has just arrived from abroad to take a special course aided by scholarships given by the League of Red Cross Societies at Geneva. They will live at one of the college hostels, and be prepared, theoretically and practically, for public-health work in their own countries. Among the countries represented are Peru, Venezuela, Belgium, Denmark, France, Greece, Italy, Poland, Roumania, Russia, Czecho-Slovakia, Serbia, and Switzerland. Miss Waters, the assistant of Miss Fitzgerald, who is director of the Nursing Division of the League, will be in residence with these ladies, who all speak English. No doubt they will receive hospitality in many directions, and we trust they may carry back none but happy memories of their year in England. Dr. Janet Lane-Claypon, Dean of the King's College Department at Campden Hill, is to be warmly congratulated on the services she is rendering to the nursing profession by the courses so successfully arranged for nursing sisters.

---

The French War Emergency Fund, which did such admirable work in undertaking invalid cookery in many French military hospitals and providing medical and other comforts for the French wounded, found itself with £10,000 in hand on the cessation of its labours. This has been devoted to founding a cottage hospital and welfare centre at Anonville, close to the devastated area. Eight beds will be available, and the intention is to fully equip and partially endow the institution. The money could hardly have been dedicated to a better use. The little hospital will be a centre whence the benefits of skilled nursing can radiate to a wide district, and will confer a great boon upon the town.

---

A Red Cross nurse was fined £20 at Matlock recently for stealing two purses from the cloak-room of a hydropathic establishment while the guests were at luncheon. She pleaded guilty, and the magistrate, on hearing her story, inflicted a fine in lieu of imprisonment. It was stated by her solicitor that she was the wife of a Russian merchant and was in Petrograd when war broke out. She joined the Red Cross as a nurse and distinguished herself by saving the lives of two Russian officers at the front, for which she obtained the curious decoration of a general's epaulette. Subsequently, after a painful experience in a bombed hospital, she developed symptoms of shell-shock and neurasthenia, which rendered her irresponsible for her actions. During the revolution she had been compelled to work in a hospital under Bolshevik rule "to save her life."

---

In London the scarcity of probationers is beginning to be less acutely felt. In more than one of the leading training-schools we are informed that during the last few weeks excellent candidates have presented themselves, and that all probable vacancies have been filled well into next year. This is a very good sign. The natural flow of candidates worth having is from the top downwards, and when the major hospitals begin to have an overflow of candidates it may safely be predicted the others will soon participate in the healing stream.

---

A distinct honour has been paid to the memory of Dr. John Leake, who was largely instrumental in founding the General Lying-in Hospital, York Road; in the decision to re-name that road Leake Street. Dr. Leake, who died in 1792, was an eminent writer on the diseases of women, and in 1765, mainly through his exertions, the lying-in hospital was opened in Westminster Bridge Road. In 1829 it was removed to its present position, and a year later was incorporated. The hospital was principally intended as an asylum "for the wives of poor industrious tradesmen and distressed householders, who, either from unavoidable misfortunes, or from the burden of large families, are reduced to want and rendered incapable of bearing the expenses incident to the lying-in state, and also for the wives of indigent soldiers and sailors." "But the governors, in the spirit of true philanthropy," writes one historian, "have extended the benefits of the institution to unmarried females, restricting this indulgence, however, to the first instance of misconduct."

---

The General Committee of the Sunderland Royal Infirmary has decided to reduce the age of admission for probationers from twenty-one to nineteen years.

